# *CCAT2* is an oncogenic long non-coding RNA in pancreatic ductal adenocarcinoma

**DOI:** 10.1186/s40659-017-0149-0

**Published:** 2018-01-03

**Authors:** Yi Cai, Xiaomei Li, Peng Shen, Dong Zhang

**Affiliations:** 1 Department of Geriatric Oncology, The General Hospital of Chinese People’s Liberation Army, 28 Fuxing Road, Haidian District, Beijing, 100853 People’s Republic of China; 2 The Fourth Division of Department of Internal Medicine, Huailai County Hospital, Fuqianddong Rd, Huailai, Zhangjiakou, Beihe, 075400 People’s Republic of China

**Keywords:** PDAC, lncRNA CCAT2, KRAS, MAPK signaling

## Abstract

**Background:**

Pancreatic ductal adenocarcinoma (PDAC) is highly aggressive with poor prognosis. Long non-coding RNAs (lncRNAs), a group of non-coding RNAs, play important roles in the progression of PDAC. This study aimed to investigate the potential involvement of lncRNA *CCAT2* in PDAC tumorigenesis.

**Methods:**

Expression of *CCAT2* was detected by quantitative real-time PCR (qRT-PCR) in 80 human PDAC tissues and three PDAC cell lines. The effects of *CCAT2* silencing in PANC-1 cells on cell proliferation and invasion were studied using MTT assay and transwell assay, respectively. The effect of *CCAT2* silencing on tumorigenesis was assessed by PANC-1 xenograft in vivo. Using si-KRAS, the role of KRAS to regulate *CCAT2* was evaluated by qRT-PCR and luciferase reporter assay. The involvement of MEK/ERK and PI3K/AKT signaling in *CCAT2* regulation was investigated by pathway inhibitors PD98059 and LY294002, respectively.

**Results:**

*CCAT2* was significantly elevated in high-grade PDAC tissues and higher *CCAT2* expression was correlated with lower survival rate in PDAC patients. *CCAT2* was up-regulated in PDAC cell lines, as compared with normal pancreatic cells. Silencing of *CCAT2* inhibited cell proliferation and invasion in PANC-1 cells in vitro, and attenuated tumorigenesis of PANC-1 xenograft in vivo. Furthermore, *CCAT2* was regulated by KRAS through MEK/ERK signaling pathway.

**Conclusions:**

*CCAT2* is an oncogenic lncRNA in PDAC likely regulated by the KRAS-MEK/ERK pathway. It could be a potential diagnostic biomarker and therapeutic target for PDAC.

## Background

Pancreatic ductal adenocarcinoma (PDAC), which derives from the epithelial cells of pancreatic duct, is the predominant form of pancreatic cancer [1, 2]. Despite tremendous efforts to understand the pathogenesis and to improve diagnostic and therapeutic strategies of PDAC, it remains to be an incurable lethal disease with less than 5% of overall 5-year survival rate.

More than 90% of PDAC carry activating *KRAS* mutations, which are initiating genetic alterations of this disease [3]. The *KRAS* proto-oncogene encodes for the KRAS protein. KRAS, as a small GTPase, couples various growth-factor receptors on the cell membrane to intracellular signaling pathways and transcription factors, thus controlling diverse cellular processes [4]. Most *KRAS* mutations impairs intrinsic GTPase activity of KRAS, resulting in an aberrant protein that is constitutively activating downstream oncogenic signaling pathways, including PI3K/AKT and MEK/ERK [5]. Meanwhile, aberrant KRAS constantly activates a wide range of transcription factors, promoting cell proliferation, survival, transformation, adhesion, and migration [6]. Although KRAS signaling is perceived as the major driving force of PDAC [7], intensive effort to explore KRAS as effective therapeutic target in PDAC has largely failed to reach the clinic [8]. Therefore, there is an urgent need to develop alternative strategies to effectively target KRAS signaling, such as blocking KRAS downstream pathways, KRAS downstream effectors or KRAS upstream modulators.

Recent studies have suggested that long non-coding RNAs (lncRNAs) (> 200 nucleotides in length), one of the two widely-investigated classes of non-coding RNAs, plays essential roles in the development, progression, drug resistance, and epigenetic modification of PDAC [9]. Importantly, several lncRNAs closely correlate with KRAS signaling in PDAC. For instance, lncRNA *MIR31HG* competes with *KRAS* for *miR*-*193b* binding site, therefore abolishing the inhibitory effect of *miR*-*193b* on KRAS and promoting PDAC progression [10]. lncRNA *MALAT1* could sequester *miR*-*217* via direct binding, thus protecting KRAS from *miR*-*217*-mediated degradation and inducing PDAC proliferation [11, 12]. Nevertheless, the underlying mechanisms of lncRNA-mediated regulation in KRAS signaling or PDAC remain unclear.

LncRNA *CCAT2*, located at 8q24, is recently identified from microsatellite-stable colorectal cancer. It induces tumor growth, metastasis, chromosomal instability, and is considered as an oncogenic lncRNA [13]. Further studies confirmed the involvement of *CCAT2* in the tumorigenesis of many other cancers, including cervical cancer [14, 15], bladder cancer [16], ovarian cancer [17], hepatocellular carcinoma [18], glioma [19], gastric cancer [20] and breast cancer [21]. Here, we aimed to investigate the expression of *CCAT2* in human PDAC tissues and PDAC cell lines, to determine the functions of *CCAT2* in PDAC in vitro and in vivo, and to explore the involvement of *CCAT2* in KRAS signaling in PDAC.

## Methods

### Human PDAC tissues collection

A total of 80 PDAC patients under pancreaticoduodenal resection were enrolled from The General Hospital of Chinese People’s Liberation Army between March 2007 and October 2015. Clinical characteristics of these patients were summarized in Table 1. The resected PDAC tissues were fixed in formalin and embedded in paraffin for pathological diagnosis, or snap-frozen immediately in liquid nitrogen for RNA extraction.

**Table 1:** Correlations between *CCAT2* expression and clinicopathologic variables in 80 cases of human PDAC

Characteristics	No. of patients	*CCAT2* expression [case (%)]		*P* value
		Low	High	
Age (years)				
< 60	43	48.8	51.2	0.426
> 60	37	42.2	56.8	
Gender				
Male	47	53.2	46.8	0.411
Female	33	42.4	57.6	
Tumor size (cm)				
< 4	31	61.3	38.7	0.078
≥ 4	49	44.9	55.1	
Histologic grade				
G1 or G2	35	68.6	31.4	0.004
G3	45	26.7	73.3	
pT category				
T1 or T2	31	67.7	33.3	0.007
T3 or T4	49	20.4	79.6	

### HE staining

The pathological tissues were fixed in normalized fixative, consisting of 4% paraformaldehyde in 0.01 M phosphate-buffered saline, overnight at room temperature. The tissue blocks were then dehydrated with an ascending ethanol series, cleared with xylene and then embedded in paraffin. The paraffin blocks were cut into transverse serial sections of 10 μm thickness. Next, five sections from each animal were randomly chosen and mounted on poly-l-lysine coated slides for HE staining. The detailed protocol was shown below: (1) Deparaffinize sections, 2 changes of xylene, 10 min each. (2) Re-hydrate in 2 changes of absolute alcohol, 5 min each. (3) 95% alcohol for 2 min and 70% alcohol for 2 min. (4) Wash briefly in distilled water. (5) Stain in Harris hematoxylin solution for 8 min. (6) Wash in running tap water for 5 min. (7) Differentiate in 1% acid alcohol for 30 s. (8) Wash running tap water for 1 min. (9) Bluing in 0.2% ammonia water or saturated lithium carbonate solution for 30 s to 1 min. (10) Wash in running tap water for 5 min. (11) Rinse in 95% alcohol, 10 dips. (12) Counterstain in eosin-phloxine solution for 30 s to 1 min. (13) Dehydrate through 95% alcohol, 2 changes of absolute alcohol, 5 min each. (14) Clear in 2 changes of xylene, 5 min each. (15) Mount with xylene based mounting medium.

### Cell lines and chemicals

Human pancreatic cancer cell line PANC-1, SW1990, PC-3 and human normal pancreatic ductal epithelial cell line HPDE6-C7 were purchased from Beijing Zhongyuan Ltd. (China). Cells were maintained in DMEM supplemented with 10% fetal bovine serum (Gibco, Life Technologies, Carlsbad, CA, USA) at 37 °C under 5% CO_2_ in a humidified incubator. The MEK/ERK signaling inhibitor PD98059 [22] and PI3K/AKT signaling inhibitor LY294002 [23] were purchased from Sigma Ltd. (Shanghai, China).

### siRNA transfection

All siRNAs were commercially constructed by Shanghai GenePharma Co. Ltd (Shanghai, China) and transfected with Lipofectamine RNAiMAX reagent (Thermo Fisher Scientific, Sunnyvale, CA, USA) according to the manufacturer’s protocol. Sequences for siRNA targeting *CCAT2* (si-CCAT2) were 5′-GUGCAACUCUGCAAUUUAAUU-3′ (S) and 5′-UUAAAUUGCAGAGUUGCACUU-3′ (AS); Sequences for siRNA targeting *KRAS* (si-KRAS) were 5′-AUAUUCAGUCAUUUUCAGCAG-3′ (S) and 5′-GCUGAAAAUGACUGAAUAUAA-3′ (AS). A scramble siRNA (Scramble) was used as negative control, the sequences for Scramble were 5′-GUAAUUUAAGCAACUCUGCUU-3′ (S) and 5′-UUAGUUGCACAAAUUGCAGUU-3′ (AS)

### RNA extraction and quantitative RT-PCR

RNA isolation, reverse transcription and qRT-PCR were performed as described previously [21] with minor modification. Total RNA was isolated using TRIzol reagent (Thermo Fisher Scientific, Sunnyvale, CA, USA), and reverse transcribed with SuperScript First Strand cDNA System (Thermo Fisher Scientific, Sunnyvale, CA, USA) according to the manufacturer’s instructions. qRT-PCR was performed on a 7300 Real-Time PCR System (Thermo Fisher Scientific, Sunnyvale, CA, USA) using SYBR green agent (Applied Biosystem, Foster City, CA, USA). The cycling conditions were 40 cycles of 94 °C for 30 s, 60 °C for 30 s and 72 °C for 30 s. The primers were: *CCAT2* forward (5′-AGACAGTGCCAGCCAACC-3′) and reverse (5′-TGCCAAACCCTTCCCTTA-3′); *GAPDH* (internal control) forward (5′-ACCCAGAAGACTGTGGATGG-3′) and reverse (5′-TCAGCTCAGGGATGACCTTG-3′).

### MTT assay

PANC-1 cells were seeded into 96-well plates at a density of 5 × 10^3^ cells/well and a final volume of 150 μL/well in triplicate per experiment. After 0, 24, 48, 72 or 96 h, MTT reagent (Sigma-Aldrich, Shanghai, China) (20 μL) was added and cells were incubated for 4 h at 37 °C. The medium was then discarded and cells were oscillated in 150 μL/well dimethyl sulfoxide for 15 min. The absorbance was measured at 490 nm using a Fluoroskan Ascent FL Microplate Fluorometer (Thermo Scientific, Sunnyvale, CA, USA).

### Transwell assay

Cell motility was evaluated with the 24-well Boyden chamber with 8-μm pore size polycarbonate membrane (Corning Incorporated, Los Angeles, CA, USA) with matrigel (BD Biosciences, San Diego, CA, USA) to simulate matrix barrier. 48 h after siRNA transfection, 4 × 10^3^ cells in 200 μL serum-free DMEM medium were seeded on top of the transwell membrane in the upper chamber. 600 μL of DMEM medium containing 20% fetal bovine serum or equal volume of migration buffer was added in the lower chamber as chemo-attractant or negative control, respectively. After 24-h incubation, the membranes were fixed with methanol and stained with 0.1% crystal violet at 37 °C. Three visual fields were randomly selected from each membrane, and the number of migrated cells were counted underneath an inverted microscope.

### Tumor xenograft model

Female athymic nude mice (4–5 weeks old) were purchased from Vital River Laboratory Animal Technology Ltd. (Beijing, China). 2 × 10^6^ PANC-1 cells, transfected with si-CCAT2 or Scramble, were mixed with equal volume of matrigel (BD Biosciences, USA) and injected subcutaneously into the right flank of nude mice to establish PDAC xenograft models [21]. The perpendicular diameters of all tumors were measured once a week with a digital caliper and the tumor volumes were calculated as (length × width^2^)/2. All mice were sacrificed at the end of week 4 to compare tumor growth. The animal protocol was approved by the Institutional Animal Care and Use Committee of the General Hospital of Chinese People’s Liberation Army.

### Western blotting

Cell lysates were harvested and protein concentrations were determined via bicinchoninic acid protein quantification method [24]. 40 μg of total protein were separated by SDS-PAGE gel electrophoresis and electrotransferred onto PVDF membranes. Primary rabbit antibodies against total ERK (No. 4695), p-ERK (No. 4370), total AKT (No. 4685), p-AKT (No. 4060), KRAS (No. 3339) (Cell Signaling Technology Inc., Berkeley, CA, USA) and GAPDH (No. TA-08) (loading control) (Zhongshanjinqiao Biotech, China) were incubated at 4 °C overnight at a dilution of 1:1000, and after washed with PBST for three times, the secondary horseradish-peroxidase-labeled antibody was incubated at room temperature for 2 h at a dilution of 1:5000. Finally, the relevant protein was visualized by staining with the enhanced chemiluminescent (ECL) kit (Haigene, Harbin, China). The relative levels of each target protein to the control (total AKT, total ERK, or GAPDH) were determined using a UVP bioimaging system and LabWorks 4.6 software (UVP, Upland, CA, USA).

### Luciferase assay

PANC-1 cells in 24-well plates were cotransfected with a dual-luciferase reporter plasmid containing *CCAT2* promoter-reporter plasmid (Genechem Ltd., Shanghai, China), in combination with si-KRAS or Scramble for 48 h. The luciferase activity was measured using the Dual-Lucy Assay Kit from Vigorous Biotechnology (Beijing, China) according to the manufacturer’s protocol. All transfections were repeated for at least three times.

### Statistical analysis

All statistical analyses were carried out using SPSS 17.0 (SPSS Inc., USA). The overall survival was evaluated by the Kaplan–Meier method. All other data were analyzed using independent two-tailed Student’s *t* test. Data shown were mean ± SEM or representative of at least three independent biological repeats. P < 0.05 was considered as statistically significant.

## Results

### *CCAT2* is upregulated in PDACs and predicts patients with poor survival

**Fig. 1:**
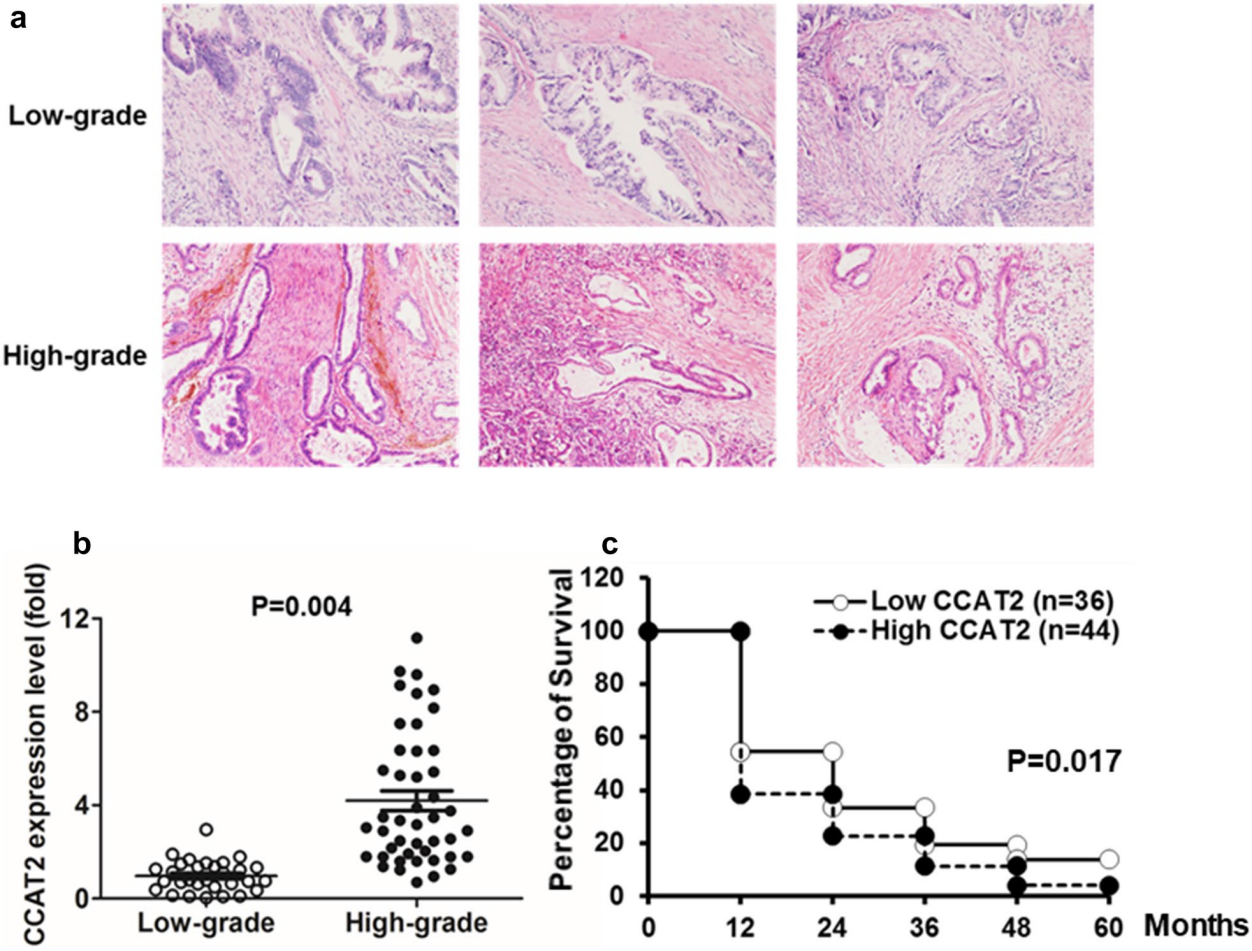
*CCAT2* is upregulated in human PDAC and predicts patients with poor survival. **a** HE staining to characterize histologically low-grade and high-grade human PDAC tissue samples. One randomly-selected field from three randomly-selected patients in each group were shown. **b** *CCAT2* expression was compared by qRT-PCR between low-grade (n = 35) and high-grade (n = 45) PDAC samples (P = 0.004). **c** Kaplan–Meier survival curves stratified a total of 80 PDAC patients according to *CCAT2* expression. P = 0.017 between low *CCAT2* and high *CCAT2* groups

We first categorized 80 human PDAC tissue samples into low-grade (n = 35) or high-grade (n = 45) (Fig. 1a), and used qRT-PCR to detect their endogenous *CCAT2* expression. *CCAT2* levels were significantly higher in the high-grade PDAC tissues than those in the low-grade PDACs (P = 0.004) (Fig. 1b, Table 1). *CCAT2* expression was also significantly related to pT (primary tumor) categories (P = 0.007, T1 + T2 vs T3 + T4) (Table 1). However, there was no correlation between *CCAT2* expression and age, gender, or tumor size in these 80 PDAC patients (Table 1). A Kaplan–Meier survival curve showed that the overall survival rate of PDAC patients in high *CCAT2* expression group (n = 44) markedly decreased as compared with that of low *CCAT2* expression (n = 36) group (Fig. 1c).

### Silencing of *CCAT2* inhibits PDAC cell proliferation and invasion in vitro

**Fig. 2:**
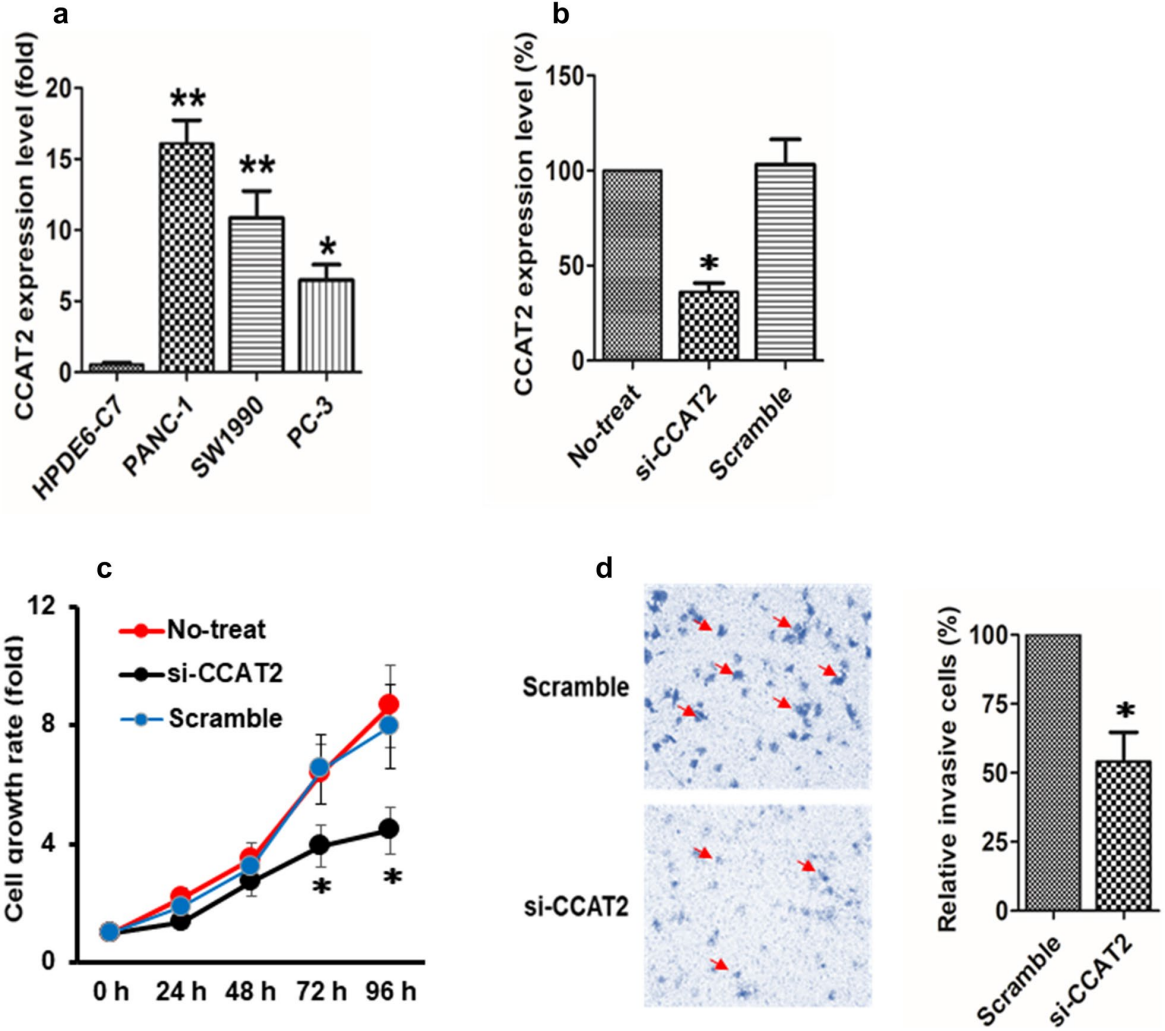
Silencing of *CCAT2* inhibits PDAC cell proliferation and invasion in vitro. **a** *CCAT2* expression was compared between HPDE6-C7, a normal human pancreatic ductal epithelial cell line, and three PDAC cell lines, including PANC-1, SW1990 and PC-3. **P < 0.01; *P < 0.05. **b** *CCAT2* expression in si-CCAT2-treated PANC-1 cells was compared to that in cells with no treatment (No-treat) or cells treated with scramble siRNA (Scramble). *P < 0.05 as compared with Scramble. **c** MTT assay detected fold change of cell growth rate in PANC-1 cells with no treatment, treated with Scramble siRNA or si-CCAT2 for 0, 24, 48, 72, and 96 h. *P < 0.05 as compared with Scramble. **d** Transwell assay evaluated cell invasion in si-CCAT2-treated and Scramble-treated PANC-1 cells. *P < 0.05. Representative images were shown on the left. From **a** to **d**, data shown were mean ± SEM of three independent biological repeats

Compared with HPDE6-C7, a normal human pancreatic ductal epithelial cell line, we found higher *CCAT2* expression in all three PDAC cell lines, including PANC-1 (P < 0.01), SW1990 (P < 0.01) and PC-3 (P < 0.05) (Fig. 2a). Next, we knocked down the expression of endogenous *CCAT2* in PANC-1 cells via *CCAT2* siRNA (si-CCAT2) to investigate its role in proliferation and invasion of PDACs (Fig. 2b). *CCAT2* silencing dramatically inhibited the proliferation of PANC-1 cells at 72 (P < 0.05) and 96 (P < 0.05) hours post-transfection (Fig. 2c). Furthermore, compared with Scramble-treated cells, the capability of invasion was significantly suppressed in PANC-1 cells transfected with si-CCAT2 (P < 0.05) (Fig. 2d).

### Silencing of *CCAT2* inhibits PDAC tumor growth in vivo

**Fig. 3:**
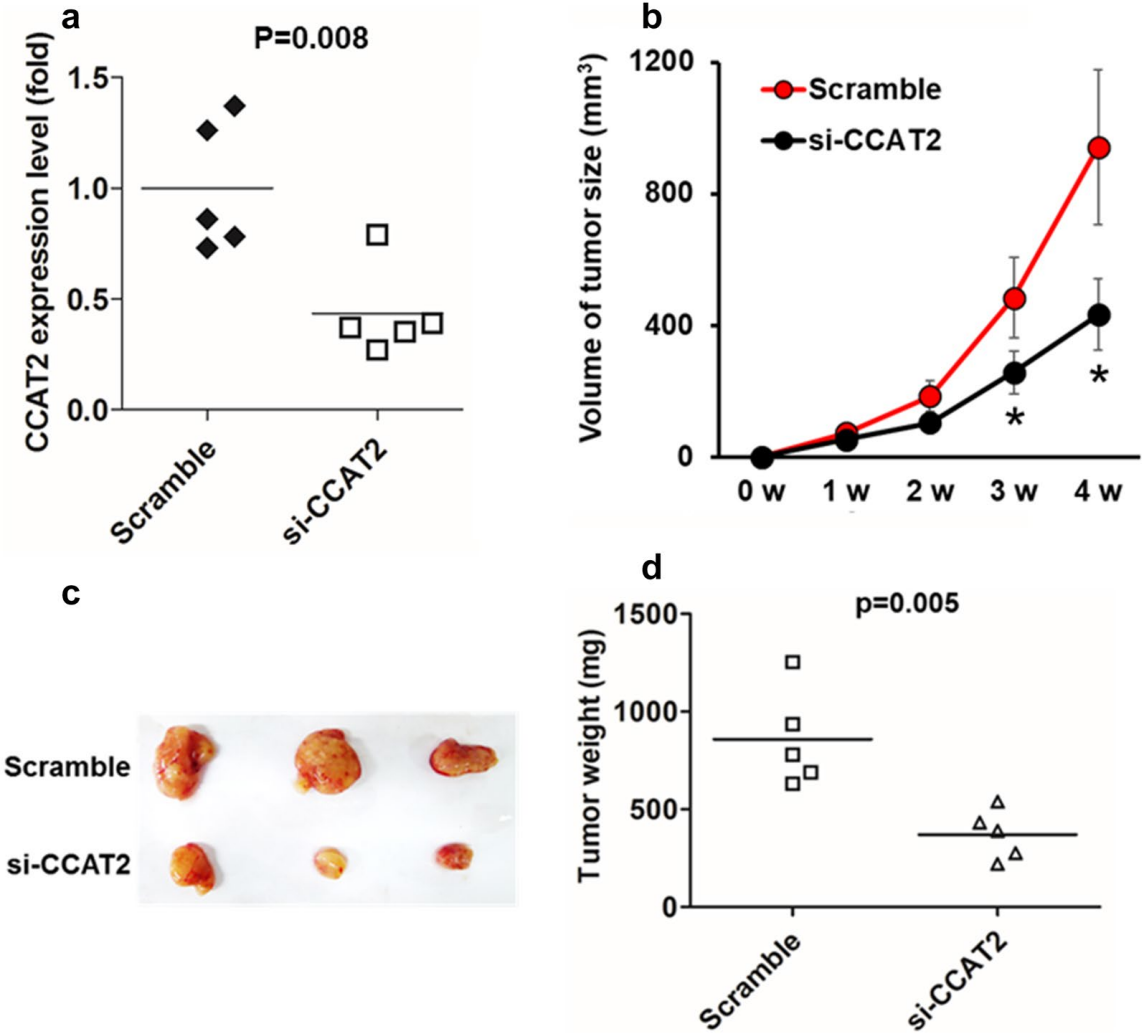
Silencing of *CCAT2* inhibits PDAC tumor growth in vivo. Female nude mice (n = 5/group) was implanted with si-CCAT2-treated or Scramble-treated PANC-1 xenografts and tumor growth was monitored continuously for 4 weeks. **a** *CCAT2* expression in xenograft PDAC tumors was compared between si-CCAT2 and Scramble groups (P = 0.008) when tumors were harvested 4 weeks after PANC-1 cell injection. **b** Tumor volume was recorded once per week after PANC-1 cells injection. *P < 0.05. **c** Representative images of PDAC xenograft tumors from each group at the time of study termination (week 4). **d** Tumor weight were compared between si-CCAT2-treated or Scramble-treated groups (P = 0.005)

To explore potential involvement of *CCAT2* in the development of PDAC, we established PDAC xenograft mouse models with PANC-1 cells transfected with either Scramble (n = 5) or si-CCAT2 (n = 5). Silencing of *CCAT2* (P < 0.01) (Fig. 3a) significantly reduced PDAC tumor growth at 3 (P < 0.05) and 4 weeks (P < 0.05) after xenograft injections (Fig. 3b). Consistently, we found tumor size (Fig. 3c) and weight (Fig. 3d) were dramatically suppressed in the si-CCAT2 group, compared with those in the Scramble group. These results suggest that *CCAT2* contributes to PDAC tumorigenesis in vivo.

### KRAS regulates *CCAT2* expression via MEK/ERK pathway

**Fig. 4:**
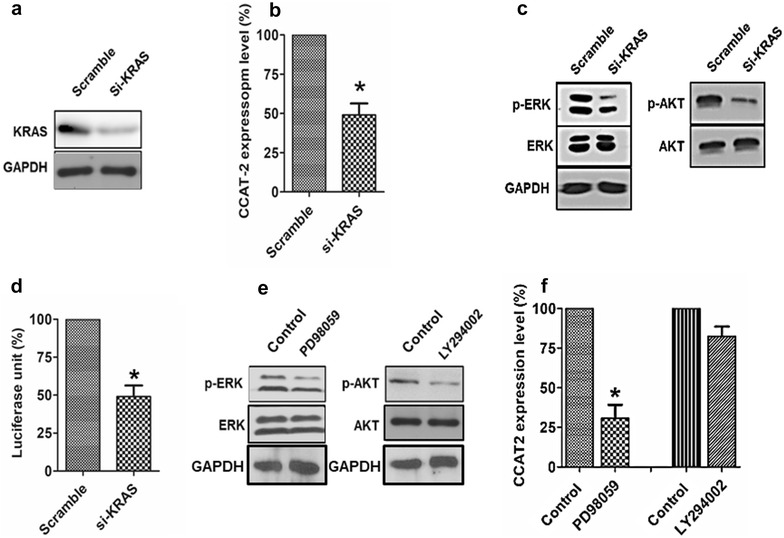
KRAS regulates *CCAT2* expression via MEK/ERK pathway. **a** The expression of KRAS was compared between Scramble- and si-KRAS-treated PANC-1 cells 48 h after transfection. **b** *CCAT2* expression in PANC-1 cells transfected with si-KRAS or Scramble. *P < 0.05. **c** Immunoblotting for p-ERK, total ERK, p-AKT and total AKT in si-KRAS-treated and Scramble-treated PANC-1 cells 48 h after transfection. **d** PANC-1 cells were co-transfected with *CCAT2* promoter-reporter plasmid in combination with si-KRAS or Scramble. Luciferase activity were measured 48 h after transfection. *P < 0.05. For **a** and **c**, GAPDH was used as loading control. Image shown was representative of three independent biological repeats. For **b** and **d**, data shown were mean ± SEM of three independent biological repeats. **e** Activation of ERK or AKT was evaluated in PANC-1 cells treated with MEK/ERK inhibitor PD98059 or PI3K/AKT inhibitor LY294002 for 24 h. **f** *CCAT2* expression in PANC-1 cells treated with PD98059 or LY294002 for 24 h. *P < 0.05. For **e** and **f**, DMSO-treated group was used as control. Image shown was representative of three independent biological repeats

Other studies have revealed that the oncogenic *KRAS* mutation is present in more than 90% of PDAC and is the driving force of pancreatic tumorigenesis [25], we showed in this study that suppression of KRAS expression (Fig. 4a) led to a significant downregulation of *CCAT2* expression in PANC-1 cells (Fig. 4b). Additionally, KRAS inhibition markedly decreased *CCAT2* promoter activity by more than 60% (Fig. 4d), indicating that KRAS might regulate *CCAT2* expression at transcriptional level. Although inhibition of KRAS attenuated the activation of both MEK/ERK and PI3K/AKT signaling pathways (Fig. 4c), we found that *CCAT2* expression was only suppressed by MEK/ERK pathway inhibitor PD98059, but not PI3K/AKT pathway inhibitor LY294002 (Fig. 4e, f). Altogether, our data indicate that KRAS might transcriptionally regulate *CCAT2* expression via MEK/ERK signaling pathway in PDAC.

## Discussion

PDAC is one of the most aggressive malignancies. Because of local invasion and distal metastasis, less than 20% of PDAC is resectable at the time of diagnosis [26]. Lack of effective biomarkers in PDAC further compromises the early diagnosis and treatment of this disease. Increased expression of *CCAT2* has been found in a wide range of cancers, promoting tumor growth, cell cycle progression, migration, invasion, metastasis, and inhibiting apoptosis [13–21], however, the function of CCAT2 in PDAC was still unknown.

In this study, we found for the first time that lncRNA *CCAT2* was significantly elevated in human high-grade (G3) PDAC tissues, as compared with low-grade (G1 or G2) tissues. The differential expression of *CCAT2* in PDAC is not age- or gender-related. Intriguingly, *CCAT2* expression is closely correlated with mortality of PDAC patients in that higher *CCAT2* levels predicts lower survival rate. Therefore, it could be promising to explore *CCAT2* as a universal predictive biomarker in PDAC, in order to benefit patients’ quality of life and prognosis. Furthermore, using PANC-1 as a PDAC cell model, we showed that inhibition of *CCAT2* significantly decreased cell proliferation and invasion in vitro, and suppressed tumorigenicity in vivo. These data suggest that *CCAT2* is oncogenic in PDAC.

Several mechanisms underlying the tumorigenic function of *CCAT2* have been proposed. *CCAT2* could activate the transcriptional activity of Wnt/β-catenin signaling pathway and promote the translocation of β-catenin from cytoplasm to nucleus [19, 21]; *CCAT2* could induce chromosomal instability, causing aneuploidy formation [13]; *CCAT2* could up-regulate the expression of *MYC* oncogene and its microRNA target *miR*-*17*-*5p* [13]; *CCAT2* could decrease E-cadherin and increase ZEB2, Vimentin, and N-cadherin, thus stimulating epithelial-mesenchymal transition (EMT) [27]. The detailed mechanism of how *CCAT2* facilitates the development of PDAC need to be further explored in our future studies.

The expression of lncRNA is under tight control. For example, lncRNA *MEG3* is decreased by hypermethylation in its promoter [28]; lncRNA *LET* is repressed by HDAC3-mediated deacetylation in its promoter [29]; lncRNA *AK019103* contains binding sites of transcription factor NF-κB, and inhibition of NF-κB dramatically suppressed DNA damage-induced *AK019103* upregulation [30]; lncRNA *HOTAIR* is inhibited by *miRNA*-*34a* via direct binding [31]; lncRNA *UCA1* could bind to RNA-binding protein hnRNP I, which in turn stabilizes *UCA1* [32]. In this study, we revealed a novel regulatory mechanism of *CCAT2*’s oncogenic potential in PDAC. Our data support that *KRAS* oncogene induces the expression of *CCAT2* via MEK/ERK, but not PI3 K/AKT signaling. Although our data are not sufficient to identify the exact factor directly regulating *CCAT2* expression in PDAC, based on the diverse functions in each tier of KRAS or MEK/ERK signaling cascade, we speculate that they could modulate *CCAT2* through DNA methylation, histone modification, downstream transcription factors, downstream miRNAs, or post-transcriptional regulations.

In summary, elevated expression of *CCAT2* is correlated with high-grade and low survival rate in PDAC. *CCAT2* facilitates proliferation and invasion of tumor cells, thus promoting PDAC progression. *CCAT2* serves as a downstream effector of KRAS and MEK/ERK signaling, and holds potential to be a novel diagnostic biomarker and a therapeutic target in PDAC. Besides comparatively small sample size (80 PDAC patients were enrolled), another limitation of our study is that we did not compare the expression of CCAT2 between normal pancreatic and PDAC tissues. However, these will be the subject of our ongoing studies.
